# Drug Susceptibility of *Mycobacterium tuberculosis* Beijing Genotype and Association with MDR TB

**DOI:** 10.3201/eid1804.110912

**Published:** 2012-04

**Authors:** Jurriaan E.M. de Steenwinkel, Marian T. ten Kate, Gerjo J. de Knegt, Kristin Kremer, Rob E. Aarnoutse, Martin J. Boeree, Henri A. Verbrugh, Dick van Soolingen, Irma A.J.M. Bakker-Woudenberg

**Affiliations:** Erasmus University Medical Center, Rotterdam, the Netherlands (J.E.M. de Steenwinkel, M.T. ten Kate, G.J. de Knegt, H.A. Verbrugh, I.A.J.M. Bakker-Woudenberg);; National Institute of Public Health and the Environment Center for Infectious Disease Control (RIVM),; Bilthoven, the Netherlands (K. Kremer, D. van Soolingen);; World Health Organization Regional Office for Europe, Copenhagen, Denmark (K. Kremer);; Radboud University Nijmegen Medical Center, Nijmegen, the Netherlands (R.E. Aarnoutse, M.J. Boeree, D. van Soolingen)

**Keywords:** multidrug-resistant tuberculosis, MDR TB, Beijing genotype strains, antituberculosis drugs, emergence, resistance, antimicrobial resistances, Mycobacterium tuberculosis, tuberculosis and other mycobacteria

## Abstract

To determine differences in the ability of *Mycobacterium tuberculosis* strains to withstand antituberculosis drug treatment, we compared the activity of antituberculosis drugs against susceptible Beijing and East-African/Indian genotype *M. tuberculosis* strains. Beijing genotype strains showed high rates of mutation within a wide range of drug concentrations, possibly explaining this genotype’s association with multidrug-resistant tuberculosis.

The emergence of *Mycobacterium tuberculosis* resistance to antituberculosis (anti-TB) drugs is a major public health challenge that is threatening World Health Organization targets set for the elimination of TB (*1*). Approximately 500,000 cases of multidrug-resistant TB (MDR TB) are diagnosed annually, but the true magnitude of the MDR TB problem is not known because adequate laboratory tools are lacking. Multiple factors contribute to low cure rates, treatment failures, and relapses: poor-quality guidance regarding treatment, HIV co-infection, transmission of resistant forms of TB, underdeveloped laboratory services, and unavailability of alternative drug treatments. However, the evolution of *M. tuberculosis* is an additional factor that presumably fuels the worldwide problem of emerging resistance. The Beijing genotype is significantly associated with drug resistance (*2**,**3*), especially in geographic areas where prevalence of resistance to anti-TB drugs is high, and it is associated with recent TB transmission (*2**–**6*). There are also indications that the population structure of *M. tuberculosis* in areas with a high prevalence of anti-TB drug resistance is changing rapidly toward an increase in Beijing genotype strains (*2**,**6**–**8*).

The World Health Organization target rates for detecting and curing TB in Vietnam have been met; however, the rate of TB infection is not decreasing as expected (*4**,**5*). Earlier in this country, the Beijing genotype was strongly correlated with MDR TB and treatment failures (*9*). Extensive molecular epidemiologic studies showed that the Beijing and East-African/Indian (EAI) genotypes are predominating in Vietnam; each lineage causes ≈40% of the TB cases. According to the single-nucleotide polymorphism typing described by Hershberg et al. (*10*), the Beijing genotype is a representative of the modern lineage, and the EAI genotype is believed to represent an evolutionary lineage more closely related to the common ancestor of the *M. tuberculosis* complex.

We compared the in vitro activity of anti-TB drugs against susceptible Beijing and EAI *M. tuberculosis* isolates from Vietnam and determined the in vitro mutation frequency of these strains during drug exposure. We also determined time-kill kinetics of anti-TB drugs and assessed the emergence of resistant mutants and the concentration range within which resistant mutants and no susceptible mycobacteria were selected. The concentration at which resistant mutants did not emerge (the mutant prevention concentration) was also ascertained. By using this approach, we established an in vitro model for determining differences in the ability of *M. tuberculosis* strains to resist anti-TB drug treatment.

## The Study

Results of a liquid culturing system (BD BACTEC MGIT 960 System; BD Diagnostics, Sparks, MD, US) (for details, see the Technical Appendix) showed that all 5 Beijing and 5 EAI genotype strains were susceptible to isoniazid (INH), rifampin (RIF), moxifloxacin (MXF), and amikacin (AMK). MICs were determined by using the agar proportion method (*11*), which showed that ranges were small for the Beijing and EAI genotype strains: INH, 0.062–0.125 mg/L; RIF, 0.125–1 mg/L; MXF, 0.125–0.5 mg/L; and AMK, 0.5–2 mg/L. Duplicate values showed only minor differences.

**Figure 1:**
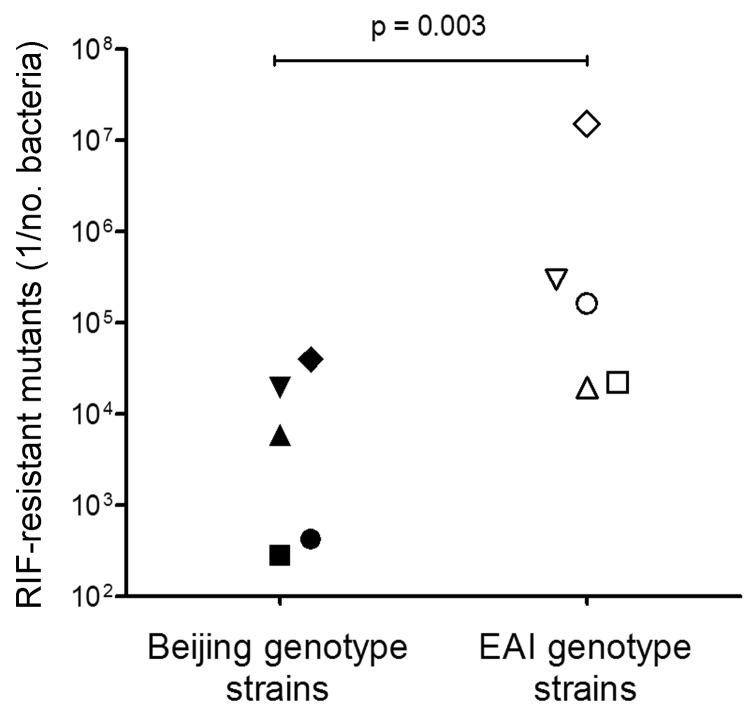
Frequency of rifampin (RIF)-resistant mutants in *Mycobacterium tuberculosis* Beijing and East-African/Indian (EAI) genotype strains (5 strains each) originating from Vietnam. Mutation frequencies were determined in duplicate. Statistical analysis was performed by using an unpaired Mann-Whitney test.

We determined the mutation frequencies of the Beijing and EAI genotype strains by using previously defined critical drug concentrations of 1 mg/L for INH, RIF, and MXF and 5 mg/L for AMK (*11**,**12*) (for details, see the Technical Appendix). The mutation frequencies of the Beijing and EAI genotype strains were similar for INH, MXF, and AMK, but they were significantly different for RIF (1.6 × 10^−5^ to 5.4 × 10^−3^ for Beijing strains vs. 6.3 × 10^−8^ to 3.8 × 10^−4^ for EAI strains; p = 0.003, unpaired Mann-Whitney test) (Table 1; Figure 1). Because rifamycin drugs are widely used to treat TB, the difference in the mutation frequencies of Beijing and EAI genotype strains for RIF is a major finding.

**Table 1:** Mutation frequency of *Mycobacterium tuberculosis* genotype strains originating from Vietnam, by antituberculosis drug

Genotype	Frequency of mutation among strains*			
	Isoniazid	Rifampin	Moxifloxacin	Amikacin
Beijing				
1585	5.7 × 10^−6^, 6.2 × 10^−6^	3.0 × 10^−3^, 4.3 × 10^−3^	4.3 × 10^−8^, 6.1 × 10^−8^	2.3 × 10^−8^, 3.2 × 10^−8^
1607	8.6 × 10^−6^, 1.4 × 10^−5^	1.5 × 10^−3^, 5.4 × 10^−3^	6.9 × 10^−8^, 2.4 × 10^−7^	8.6 × 10^−8^, 3.0 × 10^−7^
2115	7.3 × 10^−6^, 1.1 × 10^−5^	1.0 × 10^−3^, 9.2 × 10^−5^	1.0 × 10^−8^, 4.3 × 10^−8^	1.4 × 10^−8^, 2.8 × 10^−8^
2121	6.8 × 10^−5^, 2.9 × 10^−4^	2.9 × 10^−5^, 1.9 × 10^−4^	1.1 × 10^−7^, 1.6 × 10^−7^	9.3 × 10^−8^, 1.1 × 10^−7^
2145	9.1 × 10^−4^, 5.0 × 10^−4^	1.6 × 10^−5^, 5.5 × 10^−5^	7.9 × 10^−8^, 1.0 × 10^−7^	7.6 × 10^−7^, 1.1 × 10^−6^
East-African/Indian				
1627	3.7 × 10^−6^, 6.5 × 10^−6^	4.1 × 10^−6^, 2.8 × 10^−6^	9.3 × 10^−9^, 1.5 × 10^−7^	5.6 × 10^−8^, 4.5 × 10^−9^
1606	8.7 × 10^−6^, 1.6 × 10^−4^	3.8 × 10^−4^, 2.7 × 10^−5^	3.2 × 10^−8^, 1.0 × 10^−7^	7.5 × 10^−9^, 1.5 × 10^−9^
1592	1.8 × 10^−5^, 2.6 × 10^−5^	3.0 × 10^−4^, 2.4 × 10^−5^	9.9 × 10^−8^, 4.5 × 10^−8^	9.4 × 10^−8^, 1.5 × 10^−9^
1596	3.9 × 10^−5^, 2.8 × 10^−5^	1.4 × 10^−5^, 3.9 × 10^−6^	1.7 × 10^−7^, 2.0 × 10^−7^	3.7 × 10^−8^, 3.2 × 10^−7^
2113	1.3 × 10^−5^, 4.1 × 10^−5^	6.7 × 10^−8^, 6.3 × 10^−8^	1.5 × 10^−8^, 1.0 × 10^−7^	4.4 × 10^−8^, 3.3 × 10^−7^

*Determined in duplicate.

For Beijing genotype strains, the increase in mutation frequency during exposure to RIF could be due to described missense mutations in the *mut* genes (*13*). Such mutations in the *mut* genes can change the DNA repair mechanism; as a consequence, the frequency of resistant mutant formation might increase. However, a direct correlation between the occurrence of particular mutations in *mut* genes and altered mutation frequency has not been proven. Furthermore, Werngren and Hoffner (*14*) found an equal mutation frequency for Beijing (3.6 × 10^−8^) and non-Beijing (4.4 × 10^−8^) genotypes. A possible explanation for the discrepancy in findings might be the concentration of RIF used in the subculture plates. In our study, the critical concentration of 1 mg/L RIF was used (*11*), whereas Werngren and Hoffner used a concentration of 2 mg/L RIF. In addition, Werngren and Hoffner compared the Beijing and non-Beijing genotypes of several genotype families, whereas we compared Beijing and EAI genotype strains that were selected from the same tuberculosis-endemic area and during the same period.

**Figure 2:**
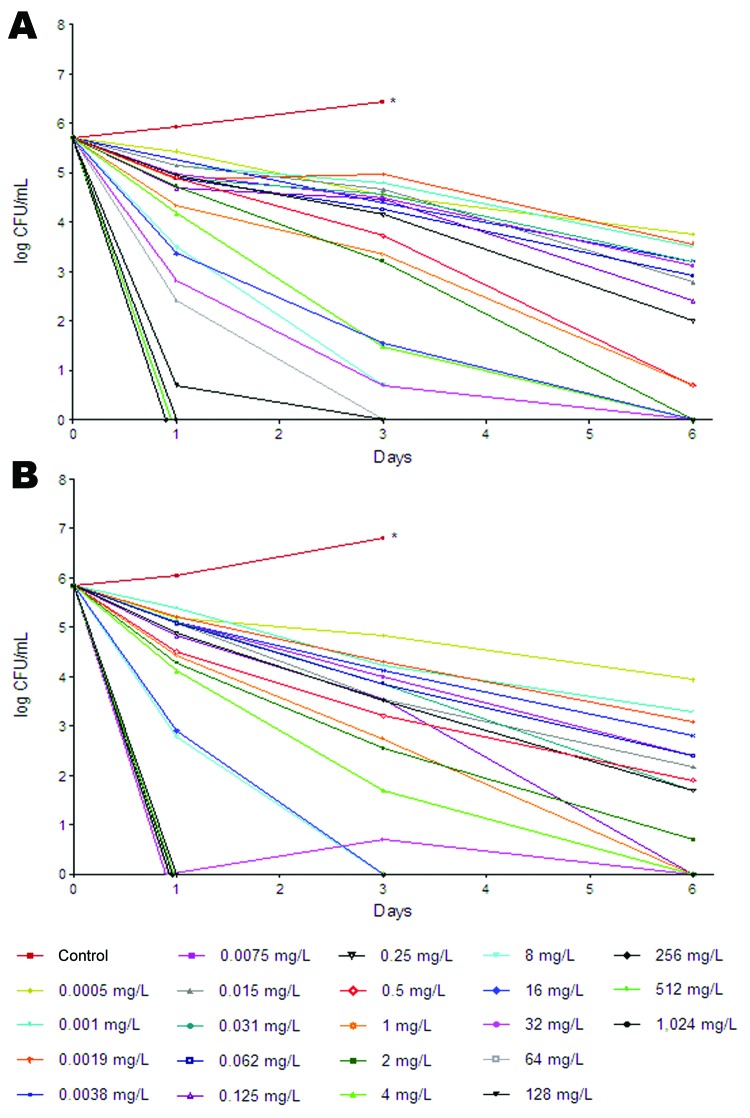
Concentration- and time-dependent bactericidal effect of rifampin (RIF) toward low-density cultures of *Mycobacterium tuberculosis* BE-1585 (5.1 × 10^5^ CFU/mL) (A) and *M. tuberculosis* EAI-1627 (6.8 × 10^5^ CFU/mL) (B). Cultures were exposed to RIF at 2-fold increasing concentrations for 6 days at 37°C. After 1, 3, or 6 days of exposure, subcultures were performed on solid media to count CFUs. *Accurate CFU counting could not be performed because complete outgrowth of mycobacteria occurred on the sixth day of RIF exposure, leading to aggregation.

We determined the time-kill kinetics of RIF toward 2 strains with significantly different mutation frequencies: Beijing-1585 (3.7 × 10^−3^ [3.0 × 10^−3^ and 4.3 × 10^−3^, duplicates]) and EAI-1627 (3.5 × 10^−6^ [2.8 × 10^−6^ and 4.1 × 10^−6^, duplicates]). Cultures with low and high densities of Beijing-1585 and EAI-1627 were investigated as described (*15*). RIF showed strong time- and concentration-dependent activity toward low-density cultures of the 2 strains (Figure 2). Low concentrations of RIF were needed to achieve >99% mycobacterial killing; differences between Beijing-1585 and EAI-1627 were minor (Table 2). However, to achieve 100% killing, especially for Beijing-1585, RIF concentrations had to be increased substantially (Table 2). Compared with the low-density culture for Beijing-1585, a substantial increase in RIF concentrations was needed to achieve 100% killing of the high-density culture (Table 2). This finding may be relevant in the clinical context because high-density mycobacteria populations are expected to exist in infected tissues of TB patients.

**Table 2:** Concentration- and time-dependent bactericidal effect of rifampin toward *Mycobacterium tuberculosis* genotypes in low- and high-density cultures*

Day	Lowest RIF concentration resulting in killing of *M. tuberculosis*, mg/L										
	Beijing-1585 genotype						EAI-1627 genotype				
	>99% killing			100% killing			>99% killing			100% killing	
	Low†	High‡		Low†	High‡		Low§	High¶		Low§	High¶
1	8	ND		256	ND		8	ND		32	ND
3	1	0.008		64	1024		0.125	0.03		8	32
6	0.001	0.008		2	64		0.001	0.06		1	2

*Cultures were exposed to RIF at 2-fold increasing concentrations for 6 days at 37°C; at indicated time-points, subcultures were performed on solid media for counting. Low, low-density culture; high, high-density culture; ND, not determined. †Density of 5.1 × 10^5^ CFU/mL. ‡Density of 4.4 × 10^6^ CFU/mL. §Density of 6.8 × 10^5^ CFU/mL. ¶Density of 3.0 × 10^6^ CFU/mL.

RIF-resistant mutants did not emerge in low-density cultures of Beijing-1585 and EAI-1627. However, RIF-resistant mutants were selected at relatively high numbers from high-density Beijing-1585 cultures compared with high-density EAI-1627 cultures. In Beijing-1585 cultures, exposure to RIF concentrations of 2–32 mg/L selected resistant mutants only; this was not observed in EAI-1627 cultures. Analysis of RIF-resistant Beijing mutants showed the following altered *rpoB* gene sequences: CAC→GAC (H526D), CAC→TAC (H526Y), and TCG→TTG (S531L), as assessed by using the GenoType MTBDR*plus* (Hain Lifescience, Nehren, Germany) assay (for details, see the Technical Appendix).

For 3 of the 4 anti-TB drugs, the difference in the range of mutant prevention concentrations for the Beijing and EAI genotype strains was small: INH, 128–256 mg/L; RIF, 256–1,024 mg/L; and MXF, 2–8 mg/L. The mutant prevention concentration for AMK was >1,024 mg/L for all strains tested.

## Conclusions

We showed that the currently used anti-TB drug susceptibility assays do not discriminate between the in vitro susceptibility, as determined by the methods used in this study, of the *M. tuberculosis* Beijing and EAI genotype strains. We also showed that the determination of mutation frequencies might be more informative than results of anti-TB drug susceptibility assays. For RIF, mutation frequencies in Beijing genotype strains were high compared with those in EAI genotype strains, and the selection of RIF-resistant mutants among Beijing strains, but not EAI strains, occurred within a wide range of RIF concentrations. In addition, the killing capacity of RIF toward the Beijing genotype is dependent on the density of mycobacteria: high concentrations of RIF are required to achieve 100% killing of high-density Beijing genotype populations but not of high-density EAI genotype populations. These in vitro characteristics might contribute to the less favorable treatment outcome of Beijing genotype TB infections and their significant association with drug resistance. Our findings demonstrate the need for anti-TB drug treatments that will prevent resistance among *M. tuberculosis* Beijing genotype TB cases, and they suggest that the development of genotype-specific TB therapy might be justified.

## Supplementary Material

Technical Appendix Detailed methods for liquid culturing system (BD BACTEC MGIT 960 System; BD Diagnostics, Sparks, MD, US) used to show drug susceptibility of Mycobacterium tuberculosis Beijing and East-African/Indian genotype strains.
