# Supplementary material for: Drug Susceptibility of Mycobacterium tuberculosis Beijing Genotype and Association with MDR TB
Source: Emerg Infect Dis. 2012 Apr;18(4):660–3. doi: 10.3201/eid1804.110912 (PMC3309663; doi:10.3201/eid1804.110912)
Supplement: Technical Appendix — Detailed methods for liquid culturing system (BD BACTEC MGIT 960 System; BD Diagnostics, Sparks, MD, US) used to show drug susceptibility of Mycobacterium tuberculosis Beijing and East-African/Indian genotype strains. [file 11-0912-Techapp_4p.pdf]

Article DOI: http://dx.doi.org/10.3201/eid1804.110912

Drug Susceptibility of *Mycobacterium tuberculosis* Beijing Genotype and Association with MDR TB

Technical Appendix

Bacteria

The 10 *M. tuberculosis* strains used in this study were all clinical isolates from Vietnam of which five represented the Beijing genotype and five the East-African-Indian (EAI) genotype. Strains were stored at the National Tuberculosis Reference Laboratory (RIVM, Bilthoven, the Netherlands) as Beijing VN 2002-1585 (Beijing-1585), VN 2002-1607, VN 1998-2115, VN 1998-2121 and VN 1998-2145, and EAI VN 2002-1627 (EAI-1627), VN 2002-1606, VN 2002-1592, VN 2002-1596 and VN 1998-2113. A subculture of strain Beijing-1585 (known as strain VN+) was previously subjected to genome sequencing described by Schürch *et al.* (Infection, Genetics and Evolution 2011; 11:587–597). The *M. tuberculosis* strains in this study were selected on basis of their diverse genotyping results, as determined by using spoligotyping and IS6110 restriction fragment length polymorphism (RFLP) typing according to the internationally standardized methods (Kamerbeek *et al.* J Clin Microbiol. 1997; 35(4):907-14 and van Embden *et al.* J Clin Microbiol. 1993; 31(2):406-9). The genotypes of the strains were defined as either Beijing or EAI according to their characteristic spoligotypes, following the well-accepted genotype definitions described by Kremer *et al.* (J. Clin Microbiol. 2004; 42(9):4040-9) and Brudey *et al.* (BMC Microbiol. 2006; 6:6-23). Comparison of the five different RFLP patterns of the five Beijing strains of this study to those of Beijing strains that were subjected to single nucleotide polymorphism (SNP) typing previously performed by Schürch *et al.* (submitted for publication) showed that strains VN 2002-1585 and VN 1998-2121 represent the typical Beijing SNP type and that strains VN 2002-1607, VN 1998-2115, and VN 1998-2145 represent an intermediate Beijing SNP type. The EAI strains showed RFLP patterns with either two (VN 2002-1596) or one copy of IS6110. However, the four 1-copy strains could be discriminated on basis of the molecular weight of the IS6110 containing RFLP fragment and/or their spoligo pattern.

Cultures

M. tuberculosis suspensions were cultured in Middlebrook 7H9 broth (Difco Laboratories, Detroit, MI, USA), supplemented with 10% oleic acid-albumin-dextrose-catalase enrichment (OADC, Baltimore Biological Laboratories, Baltimore, MD, USA), 0.5% glycerol (Scharlau Chemie S.A, Sentmenat, Spain) and 0.05% Tween 20 (Sigma Chemical Co, St. Louis, MO, USA), under shaking conditions at 96 rpm at 37°C. Vials with *M. tuberculosis* suspensions were stored at -80°C. Cultures on solid media were grown on Middlebrook 7H10 agar (Difco), supplemented with 10% OADC and 0.5% glycerol for 28-35 days at 37°C with 5% CO_2. The exact incubation time was dependent on the growth rate of the *M. tuberculosis* genotype strain investigated.

Anti-TB Drugs

The anti-TB drugs assayed were isoniazid (INH, Hospital Pharmacy; Rotterdam, The Netherlands), rifampicin (RIF, Rifadin®, Aventis Pharma B.V, Hoevelaken, The Netherlands), moxifloxacin (MXF, Avelox®, Bayer Schering Pharma A.G, Berlin, Germany) and amikacin (AMK, Hospira Benelux BVBA, Brussels, Belgium). Dilutions of the drugs were prepared according to the recommendations of the manufacturers.

Susceptibility Testing (MGIT)

The *M. tuberculosis* genotype strains were subjected to the MGIT drug susceptibility testing at the National Tuberculosis Reference Laboratory using the BD BACTEC MGIT 960 System (BD Diagnostics, 61 Sparks, MD, US) (for details, see the online Technical Appendix, wwwnc.cdc.gov/EID/pdfs/11-0912-62 Techapp.pdf) for INH, RIF, AMK and MXF susceptibility (Woods *et al.* National Committee for Clinical Laboratory Standards. CLSI document M24-A; 2011).

Minimal Inhibitory Concentration (MIC)

To determine the MIC of the *M. tuberculosis* genotype strains we used the agar proportion method as described by the Clinical and Laboratory Standards Institute (CLSI) (Woods *et al.* National Committee for Clinical Laboratory Standards. CLSI document M24-A; 2011). Colonies grown on solid media were suspended in broth using glass beads and vortexing during 4 min. The suspension was left for 30 minutes, after which the supernatant was taken and

set to an optical density of McFarland standard 1. Using broth, a 1:10 dilution of this *M. tuberculosis* suspension was plated onto solid media containing serial, twofold dilution concentrations of anti-TB drug. After incubation the degree of growth was assessed. The MIC was defined as the lowest concentration of anti-TB drug that resulted in >99% growth inhibition. MIC determinations were performed in duplicate.

Mutation Frequency (MF)

Determination of the MF of the *M. tuberculosis* genotype strains was performed using the “critical concentrations” of the anti-TB drug, being 1 mg/L for isoniazid, 1 mg/L for rifampin, 5 mg/L for amikacin and 1 mg/L for moxifloxacin, as defined by the CLSI (Woods *et al.* National Committee for Clinical Laboratory Standards. CLSI document M24-A; 2011) and Gumbo *et al.* (Antimicrob Agents Chemother. 2010; 54(4):1484-91). Starting from a high density (concentrated) *M. tuberculosis* culture (~$1x10^{10}$ cfu), serial dilutions of the *M. tuberculosis* suspension were plated onto solid media without drugs and onto solid media containing the “critical concentration” of the respective anti-TB drugs. After incubation, the total numbers of colony forming units (cfu) and numbers of resistant mycobacteria were counted and the MF was calculated. MFs were determined in duplicate. In order to assess the stability of the resistant mutants isolated from the anti-TB drug-containing solid media, 10 colonies were randomly picked and plated onto solid media without anti-TB drug. After incubation, these colonies were plated to check for re-growth on solid media containing the “critical concentration” of the same anti-TB drug.

Time-kill Kinetics

For Beijing-1585 and EAI-1627 a time-kill kinetic assay for rifampin was performed. The concentration- and time-dependent bactericidal activity of the anti-TB drug was determined as described previously (de Steenwinkel *et al.* J Antimicrob Chemother. 2010; 65(12):2582-9). In short, *M. tuberculosis* cultures at low density or at high density were exposed to rifampin at 2-fold increasing concentrations, ranging from 0,5 µg/L to 256 mg/L for six days at 37°C. On days 1, 3 and 6, samples were taken for cfu counting provided that the *M. tuberculosis* suspension did not show visible aggregation.

Selection of Drug-Resistant *M. tuberculosis*

During the time-kill kinetic assay for Beijing-1585 and EAI-1627 the selection of resistant mutants was performed. In order to detect drug-resistant *M. tuberculosis* in the low density or the high density cultures, the samples taken after six days of exposure to rifampin were cultured on rifampin-containing solid media. The concentration of rifampin in the solid media was 4-fold the “critical concentration” (4 mg/L rifampin). Resistant *M. tuberculosis* colonies, able to grow on this medium, were characterized using the GenoType® MTBDRplus assay (Hain Lifescience GmbH, Nehren, Germany), to detect the most common mutations in *rpoB* gene conferring rifampin-resistance (J Clin Microbiol. 2007; 45(8):2635-40).

Mutant Prevention Concentration (MPC)

The MPC of the *M. tuberculosis* genotype strains was determined using the protocol as described by Drlica *et al.* (J Antimicrob Chemother. 2003; 52(1):11-7) and Goessens *et al.* (J Antimicrob Chemother. 2007; 59(3):507-16). In short, from a high density (concentrated) *M. tuberculosis* culture approximately 10^{10} cfu was plated onto solid medium containing 2-fold increasing concentrations of anti-TB drug, ranging from 64 mg/L to 1024 mg/L for isoniazid, rifampin or amikacin and from 1 mg/L to 32 mg/L for moxifloxacin. The MPC was defined as the lowest concentration of anti-TB drug in the solid medium, which prevented growth of *M. tuberculosis*.
